# Feasibility of a tapering opioids prescription program for trauma patients at high risk of chronic consumption (TOPP-trauma): protocol for a pilot randomized controlled trial

**DOI:** 10.1186/s40814-019-0444-3

**Published:** 2019-05-10

**Authors:** M. Bérubé, V. Deslauriers, S. Leduc, V. Turcotte, S. Dupuis, I. Roy, S. Clairoux, S. Panic, M. Nolet

**Affiliations:** 1 Faculty of Nursing, Laval University, 1050 Avenue de la Médecine, Quebec City, QC G1V 0A6 Canada; 2 Research Center, CHU de Québec, Quebec City, QC Canada; 3 Department of Surgery, Faculty of Medicine, Université de Montréal, Montreal, QC Canada; 4 Centre Intégré Universitaire de Santé et de Services Sociaux du Nord-de-l’Île-de-Montréal, Hôpital du Sacré-Coeur de Montréal, Montreal, QC Canada; 5 Faculty of Pharmacy, Université de Montréal, Montreal, QC Canada; 6 Department of Anesthesiology, Faculty of Medicine, Université de Montreal, Montreal, QC Canada

**Keywords:** Analgesics, opioid, Opioid-related disorders, Risk factors, Secondary prevention, wound and injuries, Self-care, Cognitive therapies, Feasibility studies, Pilot projects

## Abstract

**Background:**

Opioid use disorder (OUD) and deaths related to the chronic use of opioids have increased significantly over the last two decades. Chronic consumption of opioids has been documented in many patients with traumatic injuries. Preliminary research findings have shown that interventions using cognitive-behavioral strategies were a promising adjunct in decreasing the burden associated with opioid consumption. Accordingly, the Tapering Opioids Prescription Program in Trauma (TOPP-Trauma) was developed.

**Purpose:**

To assess the feasibility of the TOPP-Trauma intervention and its research methods; and explore the potential efficacy of TOPP-Trauma in reducing opioid consumption.

**Methods:**

A 2-arm pilot randomized controlled trial (RCT) will be conducted in patients presenting a high risk for chronic opioid consumption. Fifty participants at high risk for chronic consumption of opioid will receive either TOPP-Trauma or an educational pamphlet. The feasibility assessment of TOPP-Trauma will be based on the ability to provide its components as initially planned. Several parameters will be evaluated to determine the feasibility of the research methods, including the adequacy of the sampling pool, the dropout rate, and the ease of data collection. The morphine equivalent dose (MED) per day between both groups will be measured at 6 and 12 weeks. Pain intensity and pain interference with activities will also be evaluated at the same time points.

**Discussion:**

This study will provide evidence on the feasibility of a preventive program aimed at reducing chronic opioid use in high risk trauma patients. Information will also be gathered on the methods that should be used to test the efficacy of such programs.

**Trial registration:**

International Standard Randomized Controlled Trial Number (ISRCTN): 40263056. Registered 26 May 2018.

**Electronic supplementary material:**

The online version of this article (10.1186/s40814-019-0444-3) contains supplementary material, which is available to authorized users.

## Background

The Growing Social Concern Associated with Chronic Opioid Consumption In recent years, there has been an alarming rise in chronic opioid consumption that has led to increased morbidity and mortality. The increase in opioid prescriptions, which tripled over the last decade, was identified as one of the most significant factors related to this opioid crisis [1]. Chronic opioid consumption is defined as an opioid usage persisting beyond 12 weeks [2]. Opioid consumption that exceeds this period of time will result in patients more likely to require chronic therapy for years [3] and affects up to 35% of trauma patients [4].

Chronic consumption of opioids was shown to increase the odds of opioid-use disorder (OUD), including opioid dependance, abuse, or overdose, by 15 to 122 times, depending on the dose prescribed [5]. Postsurgically, the total duration of opioid use was the strongest predictor of OUD, with each additional week of opioid use increasing the rate of OUD by 44% [6]. Similarly, between 2007 and 2017, the hospitalization rate due to opioid intoxication increased by 53% [7]. Moreover, in the United States, overdose deaths, excluding those related to fentanyl, have increased by 185% (from 6158 to 17,536 deaths) over the past 15 years [8]. In Canada, there were close to 4000 opioid-related deaths in 2017 [9]. Even more alarming, opioid-related overdoses are now one of the main causes of death for the 18-35 year old age group in the United States and Canada [10, 11].

### Risk Factors Linked to Chronic Opioid Consumption

The risk factors of chronic opioid use in trauma patients include: pre-injury use of opioids or substance abuse [2, 12–14], low socioeconomic status [13, 15], high Injury Severity Score (ISS) [15], psychological vulnerability (i.e., depression, anxiety, pain catastrophizing) [2, 16], and low pain self-efficacy (i.e., a person’s confidence in her/his ability to manage pain and perform activities while in pain) [4]. Interventions to prevent the chronic consumption of opioids among patients presenting these risk factors are therefore warranted.

### Interventions to Prevent Chronic Opioid Consumption

Secondary to the undertreatment of pain observed at the beginning of this century, the American Pain Society [17] and the Joint Comission [18] identified pain assessment as the fifth vital sign. Consequently, the widespread use of opioids became the norm, including in postoperative pain management [19]. However, until recently, opioid prescribers minimized the risks of OUD [20]. Hence, patients were, and are still, often discharged from the hospital without adequate education and follow-up [20, 21].

Fortunately, interventions using educational, cognitive (i.e., alteration of maladaptive thoughts and emotions, problem-solving) and behavioral strategies (i.e., staying active, relaxation skills, returning to preferred activities) [22] have yielded promising results. For example, a retrospective surgeon-controlled cohort study, testing the effect of preoperative counseling (i.e., providing education and advice) on the duration of postoperative opiate use in orthopedic trauma, showed that patients with counseling were significantly more likely to cease opioids by six weeks than those without [23]. However, the effect was not sustained at 12 weeks. Furthermore, a practice-based study assessing the outcomes of an acceptance and commitment therapy (ACT), which uses cognitive-behavioral strategies [24], revealed that patients who received ACT after major surgery, for reasons other than a traumatic injury, showed significantly less opioid use, pain interference with activities, and depressed mood, up to five months post-surgery [25].

Similarly, a retrospective study on patients with chronic spine pain found that those who received a combination of interventional (including exercise and physical therapy) and cognitive motivational counseling on analgesic medication used significantly less opioids at 6 months, than those who reveived interventional therapies only [26]. Cognitive motivational counseling aims to help patients rely on other pain self-management strategies, rather than on medication alone. The efficacy of such a treatment package was also underscored in a recent systematic scoping review on gastrointestinal disorders, which concluded that the greatest reductions in opioid misuse were observed when the promotion of self-management behaviors and drug monitoring with audit and feedback were used [27].

Based on positive preliminary findings associated with interventions aimed at reducing opioid consumption, we developed the Tapering Opioids Prescription Program for Trauma Patients at High Risk for Chronic Consumption (TOPP-Trauma). This program was adapted from a self-management intervention designed to prevent the acute to chronic pain transition in patients with major lower extremity trauma (iPACT-E-Trauma) [28–30]. This intervention focuses on the various dimensions of pain, pharmacological and non-pharmacological (i.e., cryotherapy, limb elevation, relaxation exercises) strategies for acute pain management, health promotion strategies, and a return to pre-injury activities. The activities included in the intervention are similar to those found in interventions based on the cognitive-behavioral approach – that is, education, problem-solving, graded activity, continued monitoring, and matching of learned self-management behaviors with real-life situations [28, 29].

iPACT-E-Trauma underwent preliminary testing in a trauma population at low risk for the chronic consumption of opioids (e.g., no pre-injury use of opioids or substance abuse, low ISS, no psychological vulnerability) [31]. Clinicians and patients gave a positive assessment of this intervention’s acceptability [28]. Furthermore, iPACT-E-Trauma was deemed feasible, [30] with achievable research methods [31].

In addition to the content and activities from iPACT-E-Trauma for the self-management of acute pain, TOPP-Trauma will integrate education about opioid misuse and patient monitoring, for those presenting risk factors for chronic opioid consumption after a traumatic injury. Before progressing to a full scale RCT, this study aims to determine the capacity to provide the components specific to TOPP-Trauma, and whether the research methods can measure the potential effects of this intervention on opioid consumption in particular.

## Methods

### Aims

The objectives of this study are to:

1. Evaluate the feasibility of TOPP-Trauma (i.e., the ability to deliver the intervention as planned and of participants to complete pre-established activities) [32].
2. Evaluate the feasibility of the research methods (i.e., the adequacy, effectiveness, and efficiency of the study protocol in gathering pertinent data from participants, that is representative of the target population and that will address the objectives preset for the intervention) [32] to test TOPP-Trauma.
3. Describe the potential efficacy of TOPP-Trauma in reducing the chronic consumption of opioid.

### Design

**Fig. 1:**
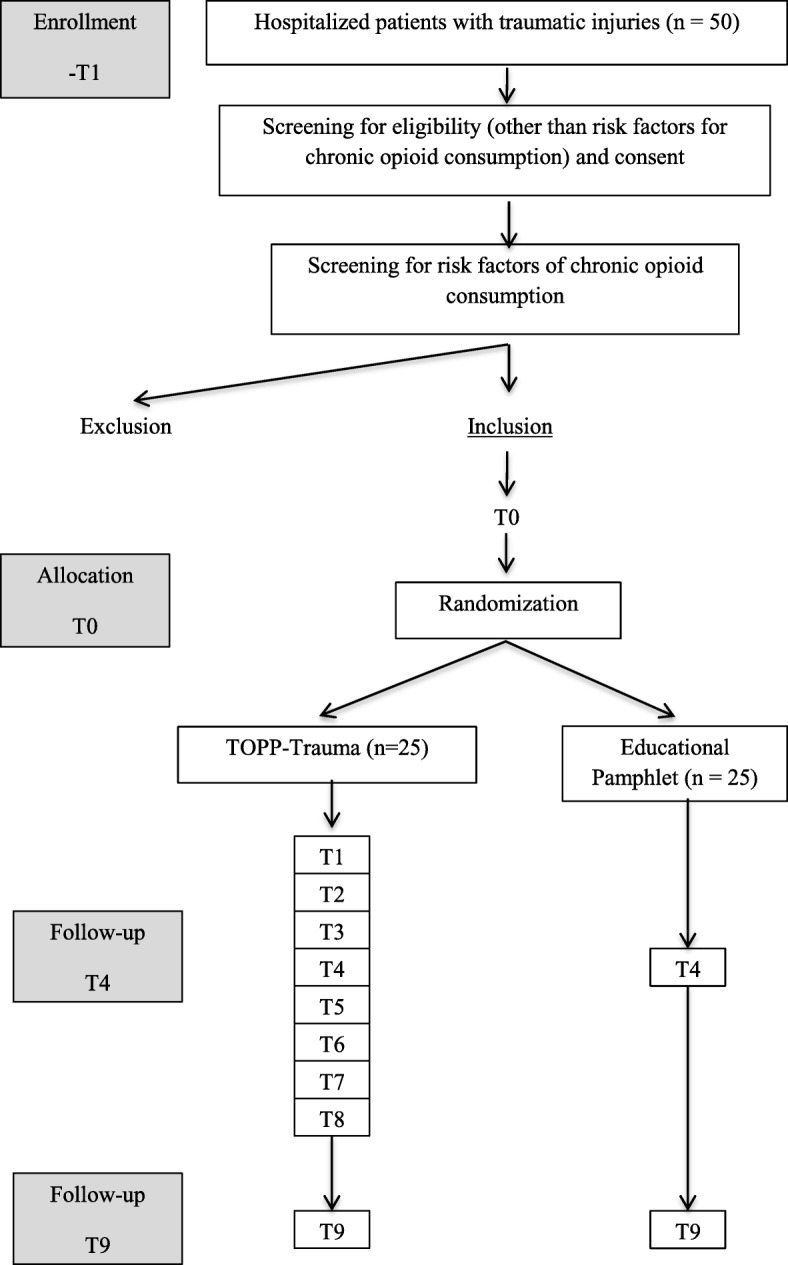
Flow diagram showing the flow of patients in the study protocol. This figure describes the process of enrollement, allocation and follow-up in relation to the intervention administration

The design selected is a two-arm pilot randomized controlled trial, to mirror the elements that would be present in a future full-scale RCT [33]. Particularly, in terms of randomization acceptance to either the experimental or control group for patients with traumatic injuries with a high risk profile for chronic consumption, and the attrition rate in both groups at the end of the study. Two groups will be studied concurrently and followed at the different study time points (T1 to T8) presented in Fig. 1. In addition to standard pain management treatments, the control group will receive an educational pamphlet, while the experimental group will receive the same pamphlet accompanied by a structured follow-up with the TOPP-Trauma research team.

### Setting

The study will be conducted in a level-1 trauma center in Montreal, Canada, admitting 1400 trauma patients, on average, annually. Ethics committee approval was obtained from the Centre Intégré Universitaire de Santé et de Services Sociaux du Nord-de-l’Île-de-Montréal Research Ethics Board (REB) (project identification number: 2019–1621).

### Eligibility Criteria

The inclusion criteria will be: 1) having suffered a traumatic injury (e.g., fractures to the extremity and the spine, thoraco-abdominal injuries), 2) at least 18 years of age, 3) able to read and speak French or English, 4) ≥ 2 doses/day of opioids during the three previous days, 5) at least one risk factor for chronic consumption, and 6) discharged directly from hospital to home. The risk factors for chronic consumption are: a) annual income ≤ $40,000 [13, 15], b) ISS ≥ 12 [15], c) pre-injury use of opioid or substance abuse [Alcohol, Smoking and Substance Involvement Screening Test – version 3.0 ≥ 11 for alcohol and ≥ 4 for other substances [2, 12–14, 34], d) anxiety or depression symptoms [scores ≥11 on the Hospital Anxiety and Depression Scale [2, 16, 35]; e) pain catastrophizing [score ≥ 20 on the Pain Catastrophizing Scale [2, 16, 36], and f) pain self-efficacy [score &lt; 17 on the Pain Self-Efficacy Questionnaire [4, 37]. The exclusion criterion will be patients with cognitive impairment [i.e., moderate-severe traumatic brain injury (TBI) – Glasgow Coma Scale score &lt; 13/15 [38], dementia and severe psychiatric disorder] affecting the capacity to participate in the study.

### Intervention

#### Control group

Participants randomized to the control group will receive the standard pain management intervention, consisting in non-opioid analgesics and opioids to control pain, as well as an educational pamphlet. The pamphlet will be provided to participants by the interventionist before hospital discharge, with no specific guided sessions, reflecting what is most commonly implemented in clinical practice. Furthermore, we hope to determine if patients receiving only an educational pamphlet are more likely to drop out than patients with follow-up. We also wish to explore if education at hospital discharge is sufficient to reduce long-term consumption of this type of analgesic.

#### Experimental group

Participants in the experimental group will receive TOPP-Trauma as well as the standard pain management intervention. The biopsychological model of pain [39], empirical data from previously tested opioid tapering interventions, the iPACT-E-Trauma intervention [28–30], and clinical knowledge on the trauma patient population guided the development of TOPP-Trauma.

#### Program Content

The biopsychosocial model of pain [39] emphasizes that disorders, such as pain, result from the dynamic interaction between biological, psychological, and social factors which may perpetuate and worsen pain. This model was influenced by the neuromatrix theory of pain [40, 41]. According to this theory, pain is the consequence of an output from a widely distributed brain neural network, impacting the biological and psychological dimensions of pain. The social dimension (e.g., daily living activities, environmental stressors) can trigger additional biopsychological reactions, thus contributing to the vicious circle of nociception, increased pain intensity, distress, and disability [42]. Hence, the biopsychosocial model of pain highlights the need to address pain management with multimodal strategies, in order to foster minimal opioid use.

TOPP-Trauma will focus on the same self-management behaviors promoted in iPACT-E-Trauma. In addition, counseling on opioid tapering and the use of alternative pain management strategies, as well as continued monitoring of opioid intake, will be provided. Recommendations on opioid tapering will be based on the adequacy of pain relief (pain intensity &lt; 4/10 and pain mildly or not interfering with daily living activities). A dose reduction of 25% per day will be suggested at each session, until opioid cessation or until patients chronically prescribed opioids reach their target maintenance dose, as per guidelines on acute pain management [43].

#### Program structure

TOPP-Trauma combines two 10-minute educational sessions within the week prior to hospital discharge, and a maximum of six 15-minute opioid tapering counseling sessions every two weeks following discharge (Table 1). A trauma case manager nurse, a nurse practitioner student or the trauma service pharmacist, who have more than five years of experience in caring for the trauma population along the acute continuum of care will provide the counseling sessions. These clinicians will receive a 4-hour training session by the principal investigator on the conceptual underpinnings of the program, the tools used in the study, and on how to deliver the program through role-play. Moreover, meetings will be held every two weeks between the PI and clinicians who will provide TOPP-Trauma until 10 patients will be recruited and until they will have provided the entire program in at least two patients.

**Table 1:** TOPP-Trauma Sessions

Sessions	Timing of Delivery	Components
Educational - 1	The week prior to hospital discharge	- The various components of pain (introduction to the biopsychosocial dimensions of pain and how they negatively or positively influence pain experience)
		- How to assess pain intensity
		- Adequate use of analgesics prescribed
		- Need to taper opioids to prevent abuse and dependence
		- How to use cryotherapy
Educational - 2	The week prior to hospital discharge	- How to use deep breathing relaxation exercises
		- The need to stay active
		- How to establish objectives for staying active with the SMART^a^ procedure (establish an objective with the participant)
		- The influence of sleep hygiene on pain and the characteristics of an adequate sleep hygiene
		- The strategies to achieve adequate sleep hygiene
Counseling - 1	One week after hospital discharge	- Assessment of patient’s average pain intensity at rest and upon movement in the last 48 h
		- Follow up on activity objective
		- Assessment of analgesics taken over the last 72 h.
		- Assessment of non-pharmacological pain management strategies used over the last 72 h and underscoring the importance of using these strategies.
		- Providing information on how to gradually reduce the consumption of analgesics (e.g., 25% opioid dose reduction OR decrease frequency of opioid use, e.g., every 6 h instead of every 3–4 h OR before activities causing high-intensity pain) if pain &lt; 4/10 and does not interfere with activities.
		- Assisting the participant to establish an objective for staying active according to the SMART procedure to be met in 2 weeks.
Counseling - 2	Two weeks after counseling session 1	- Assessment of patient’s average pain intensity at rest and upon movement in the last 48 h
		- Follow up on activity objective
		- Assessment of analgesics taken over the last 72 h.
		- Assessment of non-pharmacological pain management strategies used over the last 72 h and underscoring the importance of using these strategies
		- Providing information on how to gradually reduce the consumption of analgesics (e.g., 25% opioid dose reduction OR decrease frequency of opioid use, e.g., every 8 h instead of every 6 h OR before activities causing high-intensity pain) if pain &lt; 4/10 and does not interfere with activities.
		- Assisting the participant to establish an objective for staying active.
Counseling - 3	Two weeks after counseling session 2	- Same as counseling sessions 1 and 2.
		- Providing information on how to gradually reduce the consumption of analgesics (e.g., 25% opioid dose reduction OR decrease frequency of opioid use, e.g., every 10-12 h instead of every 8 h OR before activities causing high-intensity pain) if pain &lt; 4/10 and does not interfere with activities.
Counseling - 4, 5 and 6	Two weeks after counseling session 3, 4, 5	- Same as counseling sessions 1 to 3.
		- Providing information on how to gradually reduce the consumption of analgesics (i.e., no need to take opioids on a regular basis unless specified by her/his physician; to rely principally on acetaminophen to manage their pain and to use opioids only in the presence of pain interfering with activities not relieved by other strategies).
		- Encouraging the patient to consult her/his physician and providing a list of support resources for substance abuse if still taking opioids at counseling session 6.

^a^SMART: specific, measurable, attainable, relevant, time based

The TOPP-Trauma educational session will be based on the information included in the pamphlet given to the control group. Counseling sessions will be initiated one week after hospital discharge, and will be discontinued when patients cease opioid use or when the maximum number of planned sessions has been reached. A Program Feasibility Evaluation Logbook will guide session delivery for the trauma nurse and the trauma pharmacist. The treating surgeon will be informed of the opioid tapering plan at the time of the participants’ appointment at the outpatient clinic, to ensure treatment consistency. The number (i.e., dose) and frequency of counseling sessions was determined based on the established timeline for transitioning towards chronic opioid consumption (i.e., 12 weeks) [2] and interventions tested in other high-risk populations. These reported a significant decrease in opioid consumption after five to six sessions [25, 26]. Considering that trauma patients admitted in the trauma center where the study will be conducted come from various regions, counseling sessions will either be provided over the phone or face-to-face at the outpatient clinic, at the time of the follow-up appointment with the treating surgeon. Counseling sessions will be delivered at the outpatient clinic whenever possible in order to concomitantly inform the treating surgeons on the opioid tapering plan.

### Variables and Measurement Tools

A number of variables will be measured at different time points (Table 2) to screen patients presenting risk factor(s) for chronic opioid consumption and to meet study objectives based on the Statement for Defining Standard Protocol Items for Clinical Trials (SPIRIT) [44]. All the instruments that will be used in this study have shown adequate psychometric properties in French and English (Additional file 1) [34, 45–55]. Moreover, instruments were translated into French using a forward-backward method and/or cultural adaptation.

**Table 2:** Schedule of enrollment, intervention and assessments

Study time points											
	Enrollment	Allocation	Post-allocation^a^								Closeout
Participants timeline	-t_1_ In trauma center	0	t_1_ S1	t_2_ S2	t_3_ S3	t_4_ S4	t_5_ S5 6 weeks	t_6_ S6	t_7_ S7	t_8_ S8	t_9_ 12 weeks
*Enrollment:*											
Eligibility screen/Informed consent	√										
Screening - risk factors for chronic consumption of opioids											
- Sociodemographic questionnaire	√										
- ISS	√										
- ASSIST	√										
- HADS	√										
- PCS	√										
- PSEQ	√										
*Allocation of participants*		√									
*Intervention encounters:*											
Control group			√								
Intervention group			√	√	√	√	√	√	√	√	
*Assessments:*											
- Intervention feasibility			√	√	√	√	√	√	√	√	
- Research methods feasibility	√	√	√	√	√	√	√	√	√	√	√
- Acceptability											√
- MED/day, non-opioid analgesic(s) intake, BPI	√						√				√

^a^S1 to S8: Program sessions 1 to 8

*ISS* Injury Severity Score, *ASSIST* Alcohol, Smoking and Substance Involvement Screening Test; *HADS* Hospital Anxiety and Depression Scale, *PCS* Pain Catastrophizing Scale, *PSEQ* Pain Self-Efficacy Questionnaire, *MED* Morphine Equivalent Dose, *BPI* Brief Pain Inventory

#### Sociodemographic and clinical data

A sociodemographic questionnaire will be used to collect data on the age, sex, education, annual income, and ethnic background of participants. An Injury Profile Form will describe injury-related aspects that may affect pain intensity and recovery, including: mechanisms of injury, injuries and their grade, ISS [56], types of treatment received (surgical and nonsurgical), and number of surgeries required.

#### Screening tools

Data on annual income will be collected through the sociodemographic questionnaire. The Trauma Registry archivists will calculate the ISS within 48 h of patient recruitment or when all the diagnostic tests to identify injuries have been performed. Various instruments will be used to screen for other risk factors for chronic opioid consumption.

##### Alcohol, smoking and substance involvement screening test (ASSIST) – version 3.0

The ASSIST was developed by the World Health Organization (WHO) and an international group of addiction researchers and clinicians in response to the overwhelming public health burden associated with psychoactive substance use [57]. The ASSIST – version 3.0 is an 8-item questionnaire to screen patients at risk for substance abuse [34]. It gathers information from patients about lifetime substance use and substance use and associated problems over the last 3 months. Patients scoring between 4 and 26 (11 and 26 for alcohol) are at moderate risk of health and other problems related to substance abuse, while a score of 27 and higher suggests that they are at high risk [34].

##### Hospital anxiety and depression scale (HADS)

The HADS is a 14-item inventory divided into two subscales, each comprising 7 items, to assess anxiety (HADS-A) and depression (HADS-D) [35]. The range of each subscale is 0–21. Cut-off scores for both subscales indicate that 0–7 = normal, 8-10 = mild anxiety/depression, 11-14 = moderate anxiety/depression, and 15-21 = severe anxiety/depression [35].

##### Pain catastrophizing scale (PCS)

The PCS comprises 13 items divided into three subscales (rumination, magnification, and helplessness) measuring catastrophizing thoughts [36]. Total PCS scores range from 0 to 52; a score of 20 represents a moderate to high risk for the development of chronicity [56].

##### Pain self-efficacy questionnaire (PSEQ)

The PSEQ is a 10-item questionnaire to assess a person’s confidence in their ability to manage pain and perform activities while in pain [37]. PSEQ scores range from 0 to 49; scores &lt; 17 represent a low score preventing the modification and maintenance of behavioral change [37].

#### Feasibility and acceptability

A Program Feasibility Evaluation Logbook will make it possible to collect program feasibility data (Table 3) [58]. The Program Feasibility Evaluation Logbook will detail information on the program components that need to be delivered in each session, the pain self-management strategies applied by participants between sessions, the appropriateness of the physical environment in which the program is delivered, the time dedicated to adequately deliver the program and answer patient questions, and the challenges faced when providing the program.

**Table 3:** Variables to assess the feasibility of the intervention and the research methods

Variables	Indicators
*Intervention feasibility*	
1- The possibility to deliver the intervention as planned 2- Participants adhering to the intervention	- Provision of ≥80% of planned components of each in-presence session and of the overall intervention [59].
	- Session duration (sessions 1 to 6: 15 min).
	- Challenges faced during session delivery.
	- Attendance to ≥80% of sessions by participants [59].
	- Involvement in intervention activities and implementation of recommended self-management ≥80% behaviors by participants [59].
*Research methods feasibility*	
1- Adequacy of the sampling pool and recruitment time 2- Ease of screening 3- The possibility of applying randomization procedures as planned 4- Attrition rate in experimental and control groups 5- Ease of data collection procedures	- Obtaining consent from ≥80% of patients approached to participate in the study.
	- Percentage of eligible patients who were included in the study. - Patient’s reasons for refusal to participate in the study.
	- Difficulties in obtaining patients’ consent.
	- Recruiting study sample (i.e., 50 participants) in ≤9 months [31].
	- The time required to screen participants relative to recruitment.
	- The time required to obtain consent and baseline data relative to recruitment.
	- Eligibility criteria not limiting the pool of participants by ≥50% [32].
	- Reasons for ineligibility.
	- Difficulties when applying the randomization procedures.
	- Patient acceptance to randomization, either to the treatment or control group ≥80% of the time [32].
	- Attrition rate in experimental and control groups, i.e., ≤ 20% [60].
	- Percentage of questionnaires completed in full.
	- Pattern and rates of non-answered questions at each time measure.
	- Mean time required to complete the outcome questionnaires.
	- Mean time period between expected dates for questionnaire completion and actual completion.
	- Recall rates (telephone calls or emails) for questionnaire completion.

Gathering data on the intervention components provided and on challenges in the application of the intervention activities, in the selected mode, and the selected dose, will also demonstrate the fidelity with which the intervention can be delivered [32].

A Research Methods Feasibility Form will assess methodological research criteria parameters, established according to recognized guidelines [32, 58, 59] and data from iPACT-E-Trauma’s preliminary testing [29, 31] (Table 3). This form will monitor: (1) adequacy of the sampling pool and recruitment time, (2) ease with which participants are screened, (3) possibility of applying randomization procedures as planned, (4) attrition rate in experimental and control groups, and 5) ease of data collection (Table 2).

The Treatment Acceptability and Preference (TAP) Questionnaire [59] will be used to evaluate intervention feasibility after its completion. The TAP includes four attributes (i.e., perceived effectiveness, appropriateness, suitability, and convenience) assessed on a 5-point descriptive scale. Patients will be invited to rate intervention’s features based on these attributes. An open-ended questions was added at the end of the questionnaire to gather input on the modifications required to improve intervention acceptability. Reliability, construct validity and discriminant validity of the TAP Questionnaire have been established with a population receiving self-management intervention [60]. The intervention uptake (i.e., the implementation of self-management behaviors and the reduction of opioids as recommended), the enrollment rate, with the reasons for refusing to participate, and the attrition rate, with the reasons for dropping out, will be used to further determine TOPP-Trauma acceptability [32] for patients at high-risk of chronic opioid consumption.

### Variables and Measurement Tools

#### Morphine equivalent dose/day

Opioids taken by participants will be measured by calculating the total morphine equivalent dose (MED) per 24 h, as recommended for studies on the consumption of opioids, using the appropriate conversion methods [61, 62]. Participants will be asked to self-report their opioid use for the last three days and the day associated with the highest MED will be retained. The MED/day will be measured at 6 weeks after the beginning of TOPP-Trauma, since some studies found that a great proportion of trauma patients do not use opioids beyond this period of time [22], as well as at 12 weeks after initiating the intervention. The medical file will be consulted to determine the highest MED over three days before participant randomization, as part of baseline assessment. A pharmacist, blinded to patient allocation, will compute the MED per day.

#### Opioid delivery by the community pharmacy

A registry will be used to document the quantity of opioids prepared by the community pharmacy at 6 and 12 weeks after initiating TOPP-Trauma. Community pharmacies will be contacted to gather information on opioids delivered following participant informed consent.

#### Co-analgesia

Non-opioid analgesics will be measured by asking participants to self-report, at the various follow-up time points, on whether they used ≥2 doses of each class of analgesic, for at least one day over the last three days. The medical record will be reviewed to document the consumption of non-opioid analgesics for the 3 days before randomization. Data on non-opioid analgesics delivered by community pharmacies will also be collected at 6 and 12 weeks after beginning TOPP-Trauma.

#### Pain and function

The Brief Pain Inventory (BPI) will be used to assess pain intensity and patient function. The BPI includes 11 items: 4 on pain intensity (now, average, worst, least) measured on a 0–10 NRS (0 = no pain; 10 = worst possible pain), and 7 on pain interference with daily living activities, assessed on a 0–10 NRS (0 = does not interfere; 10 = interferes completely) [63]. The BPI item “walking” was replaced by “mobility (ability to get around)” [55] because many trauma patients may be limited in their walking capacity. Moreover, three additional items (pain interference with self-care, recreational activities, and social activities), proposed in a modified version of the BPI [55], will be added to the Pain Interference with Daily Living Activities Subscale to obtain a broader-based sample of areas that could potentially be affected by pain. The worst pain intensity upon movement, on average in the last 7 days, and the worst score for pain interference with daily living activities during the same period, will be measured at 6 and 12 weeks. Baseline measures of pain intensity and interference will cover the previous 48 h.

### Sample Size

The pilot study was not designed for adequate statistical power but to test the feasibility and potential efficacy of the program [44]. To reach these aims, we estimate that a total of 50 participants should be randomized into each group (experimental and control). This sample size is realistic considering the number of trauma patients admitted in the center where the study will be conducted.

### Recruitment

Research assistants (RA) will identify potential participants and explain the study. If patients meet the inclusion criteria and wish to participate, after receiving detailed information on what the study entails, the RA will obtain their written informed consent. A RA will then administer the screening tools to determine the presence of risk factors for chronic opioid consumption. Only patients with pre-established risk factors will be included in the study.

### Allocation

#### Randomization

The randomization sequence will be generated by a coordinating center, located within the hospital’s research center where the study will be conducted, to keep researchers blinded to allocation. A computerized random-number generator will produce the sequence (i.e., Research Randomizer). Tickets will be placed in sealed, opaque, sequentially numbered envelopes to randomize study participants to either the control or experimental group. Participants will be randomized after collecting baseline data.

#### Blinding

The expert trauma nurse and the pharmacist who will administer the program will not be blinded to group assignment. To ensure participant blinding to group assignment, the TOPP-Trauma educational session and the educational pamphlet will be given to participants in an office, in private hospital rooms, or in hospital rooms with no other trauma patients present. The RA who will collect and enter the data will be blinded to group assignment. A numerical code will be assigned to each participant, in both groups, to ensure statistician blinding.

### Data Collection

#### Procedure

At enrollment, RAs will complete the Injury Profile Form and Research Methods Feasibility Form. They will also document opioid and non-opioid consumption over the last 3 days and distribute the BPI. During program delivery, the trauma nurse and the pharmacist will record information on the feasibility of the program and the attrition rate via the Program Feasibility Evaluation Logbook and the Research Methods Feasibility Data Form. A RA will contact participants in the experimental and control groups over the phone, or meet them at the outpatient clinic, to document their opioid and non-opioid consumption and complete the BPI questionnaire, at 6 and 12 weeks after randomization. They will also contact participants’ community pharmacies to obtain information on opioid and non-opioid delivery at both time periods.

### Data analysis

#### Feasibility and acceptability

To determine the feasibility of TOPP-Trauma, the rates of program components actually delivered to participants, the challenges faced during program delivery and the pain self-management strategies applied by participants will be calculated. Moreover, mean time required for the delivery of TOPP-Trauma sessions will be computed.

Regarding the Feasibiliy of Research Methods, the following descriptive data will be obtained: 1) the number of patients screened to participate in the study, of eligible patients, and of participants included, 2) the number of inclusion and exclusion criteria that were not met, 3) the mean time required to obtain consent, to screen participants for their chronic consumption risk, and to obtain baseline data, 3) the percentage of participants who accept to be randomized to either the experimental or control group, 4) the dropout rate relative to each program session and outcome measure time point, 5) the mean time between expected dates for questionnaire completion and actual completion, and 6) the recall rates for questionnaire completion [54].

Mean scores will be calculated with regard to data collected with the TAP Questionnaire. Answers to open-ended questions, pertaining to the modifications required to improve intervention acceptability, will be group into categories according to core themes identified during content analysis.

#### Potential efficacy of the program

All outcome data will be analyzed via an intent-to-treat approach. Mean values will be calculated to determine the direction and amplitude of the differences within and between groups on the outcome measures over time. The percentage of opioid-free participants, of participants taking ≥2 doses per day of each class of non-opioid analgesic, and of participants with mild pain intensity and pain interference with activities (i.e., &lt; 4 of a 0–10 Numerical Rating Scale) [64] will be computed. Data will be analyzed at baseline, 6 weeks and 12 weeks.

### Ethical Considerations

Procedures will be implemented to ensure that the information participants provide for this study will remain confidential. All participants will be assigned unique code numbers. Consent forms will be stored separately from the data. A master list, matching the names of participants with their study identification numbers, will be kept in a locked filing cabinet separate from the data. No names or other identifying information will appear in any data that is generated. Study findings will be presented in a comprehensive form and not linked to specific participants. All hard copies of the data will be stored in a locked filing cabinet in a locked office and electronic data will be kept in a computer secured with a password. Data will be stored for 10 years and then destroyed and treated as confidential waste or deleted from the research computer.

Protocol amendments will be communicated to the REB. Finally, the program is not known to be associated with any adverse events. However, if such events should occur during the study they will be documented.

## Discussion

### Study contributions

Chronic opioid consumption has been identified in a significant number of trauma patients. To this day, no intervention with an adequate research design has been tested to provide high-level evidence-based guidance on how to address this issue. This pilot RCT will provide the required data on the feasibility and acceptability of TOPP Trauma in order to adjust the various features of this intervention before evaluating its effects. Moreover, findings will provide information on the research methods to evaluate the effect of TOPP-Trauma, while providing an adequate estimation of within and between group differences regarding the chronic consumption of opioids, setting the stage for a full RCT. Such research projects could provide trauma teams with scientific direction on how to support patients self-manage their pain while transitioning back into the community, and reduce the social burden associated with the long-term use of opioids.

### Study limitations

There is a potential social desirability bias to this study since participants will self-report their opioid use at 6 and 12 weeks. The opioid delivery records provided by the community pharmacy, close to both follow-up time points, should help counter this, validating the participants’ assertions. This procedure will also make it possible to document the total quantity of opioids taken by participants, from hospital discharge to 6 weeks after the beginning of TOPP-Trauma, and from 6 weeks to 12 weeks.

The use of an educational pamphlet only for the control group is another limitation to consider. Indeed, experimental group participants could be more inclined to reduce their consumption of opioids because of the attention they will receive from clinicians and not the intervention per se. Despite the fact that preventing the chronic opioid consumption in high risk trauma patients likely require coaching from qualified health professionals, this limitation will be acknowledged in the interpretation of findings on TOPP-Trauma potential effect. Likewise, we will plan to use a three-arm full-scale efficacy trial to account for the attention received while testing TOPP-Trauma impacts.

Finally, participants from both groups will have to answer numerous questionnaires over a 12-week period, which could induce a participation burden. Moreover, the experimental group will have to attend several program sessions. To avoid drop out and loss to follow-up, participants will complete their sessions and questionnaires at the outpatient surgical clinic while waiting to meet their surgeon, whenever possible.

## Additional file

Additional file 1: Psychometric properties of the French and English versions of measurement instruments. (DOC 37 kb)

## Abbreviations

ACT: acceptance and commitment therapy
ACT: acceptance and commitment therapy
ASSIST: Alcohol, Smoking and Substance Involvement Screening Test
BPI: Brief Pain Inventory
HADS: Hospital Anxiety and Depression Scale
ISS: Injury Severity Score
MED: morphine equivalent dose
OUD: Opioid use disorder
PCS: Pain Catastrophizing Scale
PSEQ: Pain Self-Efficacy Questionnaire
RA: Research assistants
RCT: randomized controlled trial
REB: Research Ethics Board
TBI: traumatic brain injury
TOPP-Trauma: Tapering Opioids Prescription Program for Trauma
WHO: World Health Organization
