# Supplementary material for: Feasibility of a tapering opioids prescription program for trauma patients at high risk of chronic consumption (TOPP-trauma): protocol for a pilot randomized controlled trial
Source: Pilot Feasibility Stud. 2019 May 10;5:67. doi: 10.1186/s40814-019-0444-3 (PMC6511175; doi:10.1186/s40814-019-0444-3)
Supplement: Supplementary file 1 — Psychometric properties of the French and English versions of measurement instruments. (DOC 37 kb) [file 40814_2019_444_MOESM1_ESM.doc]

Supplementary File 1. Psychometric properties of the French and English versions of

measurement instruments

|  | Reliability | Validity |
| --- | --- | --- |
| ASSIST | French:  Cronbach’s alphas for the total substance involvement and specific substance involvement ranging from 0.74 to 0.93 [45] in patients attending primary health care, psychiatric and addiction treatment facilities.  English:  Cronbach’s alphas = 0.89 for the total score and between 0.77 and 0.94 for each substance abuse involvement score [57] in patients attending specialized drug treatment facilities and primary health care facilities from 7 countries. | French:  Strongly correlated to other screening tests (r > 0.5). Cut-off scores with acceptable specificities and sensitivities [45].  English:  Strongly correlated with other screening tests (0.59 to 0.82). Moderate to strong correlations with measures of risk factors for the development of drug and alcohol problems (r = 0.48-0.76). Cut-off scores with specificities between 50% to 96% and sensitivities between 54 to 97% [57]. |
| HADS | French:  Cronbach’s alphas > 0.8 for the total score and the two subscales in HIV-infected patients [43] and in those consulting for anxiety and depression [48]. Test-retest stability at 6 months: r = 0.72 and 0.74 for total score and the two subscales [48].  English:  Cronbach’s alphas for HADS-A varied from 0.68 to 0.93 and the HADS-D from 0.67 to 0.90 in various clinical populations [49]. | French:  Two-factors loading demonstrated [47, 48] and highly correlated (r > 0.67) with other instruments measuring anxiety and depression [48].  English:  Two-factor structure and moderate to strong correlations with other common anxiety and moderate depression questionnaires (0.49 to 0.83). Specificity and sensitivity of approximately 0.80 for both the HADS-A and the HADS-D [49]. |
| PCS | French:  Cronbach’s alpha of 0.91 for total score, 0.87 for rumination, 0.57 for magnification and 0.87 for helplessness in undergraduate students during hand immersion in ice water. Test-retest stability at 2 weeks: r ≥ 0.70 [50].  English:  Test-retest at 6 weeks: r = 0.75 in undergraduate students during a cold pressor procedure [51].  Cronbach’s alpha = 0.95 in a sample of chronic pain patients [51]. | French:  Moderately correlated (r = 0.22 to 0.48) with anxiety, depression, pain intensity and pain tolerance [50].  English:  A 3-factor structure was confirmed as well as the convergent and divergent validity non-clinical and rehabilitation populations [51]. |
| PSEQ | French:  Cronbach’s alpha of 0.92 in a chronic pain population [52].  English:  Cronbach’s alpha = 0.92 and test-retest at 3 months: r = 0.73 in sample of chronic pain patients [37]. | French:  Factor analysis confirmed one factor loading and moderately correlated (r > 0.24 to 0.57) with adaptive coping strategies [52].  English:  One-factor structure and convergent as well as divergent validity demonstrated [37]. |
| BPI | French:  Cronbach’s alphas > 0.8 in cancer patients [53] and Canadian veterans suffering from chronic pain [54].  English:  Cronbach’s alpha = 0.89 in patients with cerebral palsy [54]. | French:  A two-factor structure and moderately to strongly correlated (r ≥ 0.5) with health status and quality of life in the physical domain [54].  English:  High correlation between the composite score generated from the modified BPI and pain intensity (r = 0.66) [55]. |
